# Retraction: Novel Approach to Activity Evaluation for Release-Active Forms of Anti-Interferon-Gamma Antibodies Based on Enzyme-Linked Immunoassay

**DOI:** 10.1371/journal.pone.0197086

**Published:** 2018-05-03

**Authors:** The PLOS ONE Editors

Following publication of this article [1], concerns were raised about the scientific validity of the study as well as a potential competing interest that was not declared. The *PLOS ONE* Editors discussed the concerns with the authors and consulted external experts. In light of our editorial assessment and advice received in the expert consultations, we are retracting this article due to concerns about the scientific validity of the research question, study design, and conclusions.

Specifically, we are concerned about the overall design of the study, which aims to detect effects of a reagent diluted to such a degree that the solution is not expected to contain biochemically relevant levels of antibody. The consulted experts also raised concerns about the validity and rigor of the immunoassay system used in the study. The enzyme-linked immunosorbent assay (ELISA) used was adjusted to give barely detectable signals, which renders the assay particularly susceptible to interference. In light of these issues, we consider that the article does not present sufficient or reliable evidence to support the conclusions.

In addition to these scientific concerns, the Competing Interests statement was incomplete on the published article. The Competing Interest statement read: “GS is an employee of Ab Biotechnology Limited. ESG, AAM, SAT and OIE are employees of MATERIA MEDICA HOLDING, whose company funded this study. There are no patents, products in development or marketed products to declare. This does not alter the authors' adherence to all the *PLOS ONE* policies on sharing data and materials.” The third sentence of this disclosure is inaccurate, and ought to have disclosed that OOO "NPF "MATERIA MEDICA HOLDING" markets the release-active antibody solution used in the study.

During the journal’s post-publication assessment of this work, concerns were also raised about insufficient methodological reporting in the published article that obfuscate the assessment of the study design. The authors provided additional details about methods and reagents used in response to the journal’s queries, but even in consideration of these the fundamental concerns remain about the premise of the study, the assay used, and the overall reliability of the conclusions. In light of these issues, the *PLOS ONE* Editors retract this article.

ESG, SAB, GS, SAT, and OIG do not agree with retraction and stand by the results in the article. AAM did not respond.
